# Perovskite Quantum Dot‐Enhanced Silicon Photodetectors for High‐Performance Infrared Sensing

**DOI:** 10.1002/smsc.202500170

**Published:** 2025-07-03

**Authors:** Dohun Baek, Eunseo Nam, Su Min Park, Jeongbeom Cha, Haedam Jin, Hyeongyu Kim, Jihun Lee, Kihyun Kim, Min Kim

**Affiliations:** ^1^ School of Chemical Engineering Jeonbuk National University Jeonju 54896 Republic of Korea; ^2^ Division of Electronic and Information Engineering and Future Semiconductor Convergence Technology Research Center Jeonbuk National University Jeonju 54896 Republic of Korea; ^3^ Department of Intelligent Semiconductor Engineering University of Seoul Seoul 02504 Republic of Korea; ^4^ Division of Electronic Engineering Jeonbuk National University Jeonju 54896 Republic of Korea; ^5^ Department of Chemical Engineering Center for Innovative Chemical Processes Institute of Engineering University of Seoul Seoul 02504 Republic of Korea

**Keywords:** photodetectors, quantum dots, scallop structures, short‐wave infrared, silicon‐perovskite tandems

## Abstract

Infrared photodetectors are crucial for autonomous driving, providing reliable object detection under challenging lighting conditions. However, conventional silicon‐based devices are limited in their responsivity beyond 1100 nm. Here, a scallop‐structured silicon photodetector integrated with tin‐substituted perovskite quantum dots (PQDs) that effectively extends infrared detection is demonstrated. The scallop nanowire design creates resonant light trapping, while the PQD layer enhances charge generation and transfer, especially at wavelengths above 1000 nm. Notably, the tin‐substituted PQDs improve photodetection at 1100 nm and achieve a faster response time (≈6 ms) compared with bare silicon devices. This work establishes a viable route toward high‐performance infrared sensing using perovskite‐functionalized silicon architectures, offering promising applications in autonomous vision, biomedical imaging, and industrial diagnostics.

## Graphical abstract

A high‐performance infrared photodetector integrating scallop‐structured silicon nanowires with tin‐substituted perovskite quantum dots (PQDs) is reported, enabling enhanced responsivity beyond 1100 nm. The nanowire architecture ensures resonant light trapping, while the PQDs boost charge generation and transfer. This hybrid design achieves rapid response (≈6 ms), offering strong potential for autonomous driving and near‐infrared sensing applications.

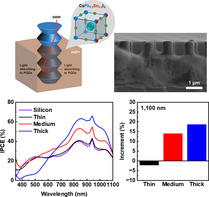

## 1 Introduction

With the growing emphasis on autonomous vehicles, substantial research investments have been directed toward enhancing photodetector technologies that bolster the safety and feasibility of autonomous driving. Although visible light is readily available in nature, it penetrates obstacles such as fog rather poorly, presenting challenges for photodetectors operating in this wavelength range.^[^ ^1^, ^2^ ^]^ As a result, the majority of photodetectors for autonomous driving are designed to operate at longer wavelengths.^[^ ^3^, ^4^, ^5^ ^]^ Currently, long‐wavelength photodetectors are frequently based on indium gallium arsenide (InGaAs), a semiconductor that absorbs light in the 800–1700 nm range.^[^ ^6^, ^7^, ^8^ ^]^ However, InGaAs remains significantly more expensive than silicon‐based alternatives.^[^ ^2^, ^9^, ^10^ ^]^ Although silicon is more cost‐effective, it intrinsically exhibits weak absorption wavelengths up to ≈1100 nm in the short‐wave infrared (SWIR) region.^[^ ^1^, ^11^ ^]^ Consequently, various strategies have been pursued to broaden the absorption range of silicon photodetectors.^[^ ^12^, ^13^, ^14^, ^15^ ^]^

Nanostructuring silicon has emerged as an effective method to enhance the interaction between incident infrared light and the photodetector.^[^ ^16^ ^]^ Conventional planar silicon photodetectors exhibit reflection losses and limited optical absorption in the SWIR region.^[^ ^17^, ^18^, ^19^ ^]^ By integrating a nanostructured surface—such as a scallop‐shaped or nanowire‐array architecture^[^ ^20^, ^21^ ^]^—light trapping is improved through mechanisms that include multiple scattering and whispering‐gallery modes. This structural modification extends the optical path length within the silicon substrate, thereby enhancing both absorption and charge carrier generation. While previous studies have investigated the impact of various structural parameters, such as the interfacial spacing between silicon nanowires,^[^ ^22^, ^23^, ^24^ ^]^ to date, a systematic investigation aimed at concurrently optimizing both light absorption and charge transfer efficiency remains lacking. Such a strategy holds significant potential for enhancing infrared photodetection performance.

One promising approach to improving the SWIR responsivity of silicon photodetectors involves combining perovskite‐based materials, notably lead–tin (Pb–Sn) perovskites, with silicon. Pb–Sn perovskite quantum dots (PQDs) are especially appealing due to their tunable bandgap, strong infrared absorption, and facile solution processability. In contrast to traditional lead‐only perovskites, Pb–Sn alloys feature a narrower bandgap, enabling the absorption of longer wavelengths in the SWIR range.^[^ ^25^, ^26^, ^27^ ^]^ Introducing tin into the perovskite framework reduces the conduction and valence band energies, thus improving infrared photon utilization. Furthermore, perovskite materials exhibit high defect tolerance and can be straightforwardly integrated onto diverse substrates. Nonetheless, issues including tin oxidation and charge extraction must be addressed to fully leverage their potential in photodetector applications.

In this work, we demonstrate a silicon–perovskite tandem photodetector by depositing tin‐substituted PQDs onto a nanostructured silicon substrate. Although lead‐based perovskites exhibit desirable optoelectronic characteristics, their large bandgap constrains their long‐wavelength absorption capabilities and lead toxicity raises environmental concerns.^[^ ^28^, ^29^ ^]^ Substituting Pb with Sn narrows the bandgap and extends infrared absorption, yet Sn oxidation remains a critical hurdle.^[^ ^30^, ^31^ ^]^ Here, we synthesize Sn‐substituted PQDs to enhance stability and infrared responsivity, and we apply them by spray coating onto scallop‐structured silicon, a configuration that boosts light trapping and charge transfer for extended SWIR detection. This approach provides a scalable, cost‐effective alternative to InGaAs photodetectors, with promising implications for infrared sensing in autonomous vision and related applications.

## 2 Results and Discussion

**Figure 1:**
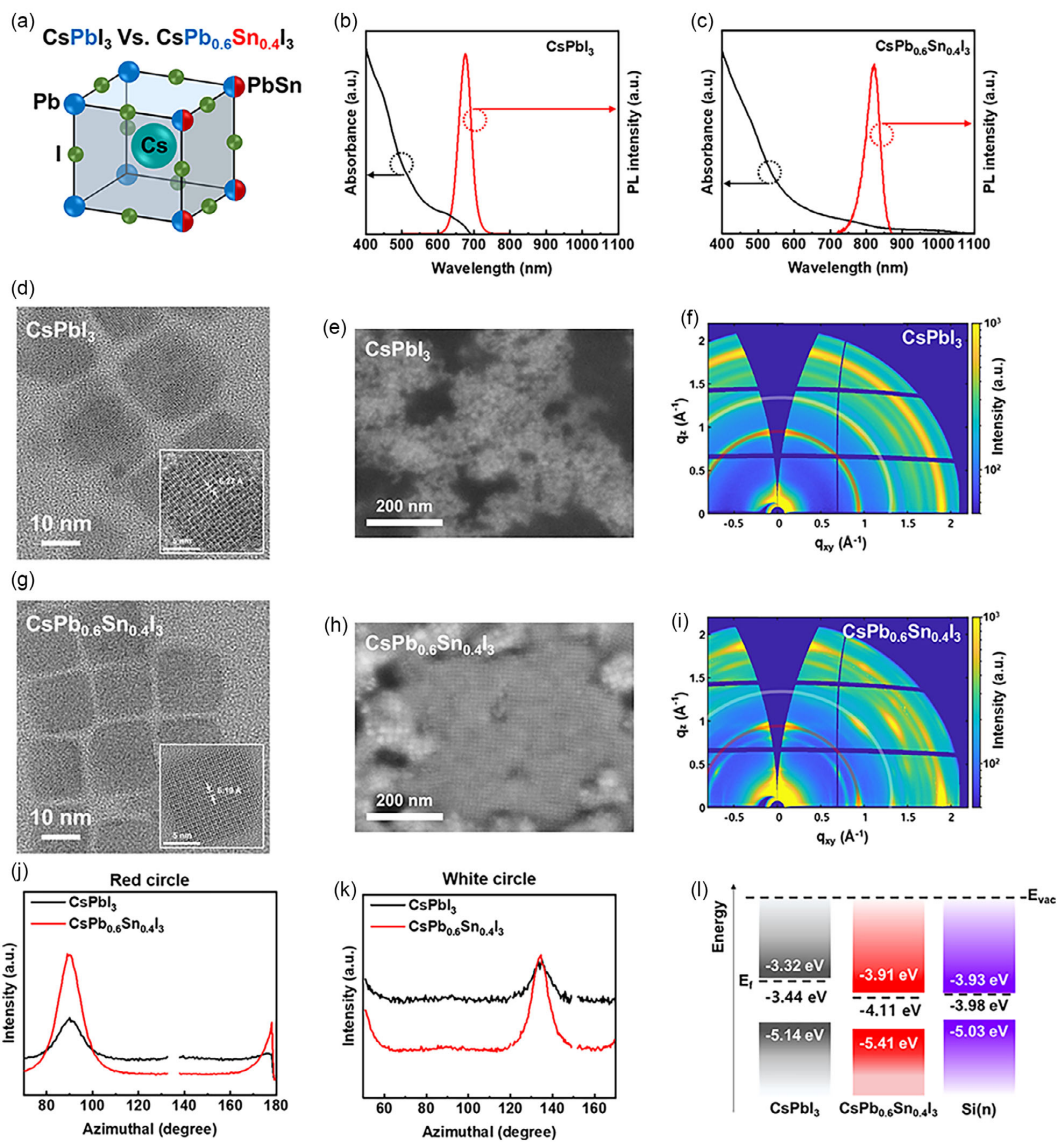
a) Crystal structure of the CsPbI_3_ and CsPb_0.6_Sn_0.4_I_3_. PL and UV‐vis spectroscopy of b) CsPbI_3_ and c) CsPb_0.6_Sn_0.4_I_3_. TEM image, SEM image, and grazing incidence wide angle X‐ray scattering (GIWAXS) of d–f) CsPbI_3_ and g–i) CsPb_0.6_Sn_0.4_I_3_. Azimuthal plot for j) (100) plane and k) (110) plane obtained from GIWAXS. l) Energy band diagram of the PQDs with Si(n)^++^.

Lead‐only PQDs (CsPbI_3_) and tin‐substituted PQDs (CsPb_0.6_Sn_0.4_I_3_) were synthesized via the hot‐injection method, as described in the Experimental Methods (**Figure** **1**a). Based on previously reported methods for measuring atomic ratio using X‐ray photoelectron spectroscopy (XPS), the atomic ratio was calculated by comparing the peak area of Pb and Sn, which confirmed a Pb‐to‐Sn atomic ratio of ≈ 6:4 in CsPb_0.6_Sn_0.4_I_3_ (Figure S1, Supporting Information).^[^ ^32^, ^33^, ^34^ ^]^

Photoluminescence (PL) and UV–vis absorption spectra (Figure 1b,c) were recorded to evaluate the optical properties of CsPbI_3_ and CsPb_0.6_Sn_0.4_I_3_. The absorption range of CsPb_0.6_Sn_0.4_I_3_ extended to 900 nm, whereas CsPbI_3_ absorbed up to 690 nm. Additionally, the PL emission exhibited a redshift from 675 nm (CsPbI_3_) to 820 nm (CsPb_0.6_Sn_0.4_I_3_), confirming enhanced long‐wavelength absorption upon Sn incorporation. Tauc plot was calculated using the following equation^[^ ^35^ ^]^

(1) $${\left(\alpha h\nu \right)}^{n}=K\left(h\nu -E_{g}\right)$$

where *α* is the absorption coefficient (2.303* A/d* with *A* being the absorbance and *d* the film thickness in *cm*), *h* is the Plank's constant, *ν* is the photon frequency, *n* is the nature of the electronic transition (*n* = 2 for a direct allowed transition), *K* is a proportionality constant, and *E*_*g*_ is the optical band gap. The bandgap of CsPbI_3_ was ≈1.82 eV, while CsPb_0.6_Sn_0.4_I_3_ exhibited a reduced bandgap of ≈1.50 eV, in line with its improved SWIR absorption properties (Figure S2, Supporting Information).

Transmission electron microscopy (TEM) images (Figure 1d,g) revealed a smaller d‐spacing for CsPb_0.6_Sn_0.4_I_3_ (6.19 Å) compared to CsPbI_3_ (6.27 Å), consistent with the smaller ionic radius of Sn relative to Pb. To assess the differences in alignment based on PQD size uniformity, we measured the size of the PQDs using TEM (Figure S3, Supporting Information). The FWHM of the CsPb_0.6_Sn_0.4_I_3_ (Avg = 14.7, σ = 0.66) was smaller than CsPbI_3_ (Avg = 10.5, σ = 1.38), indicating that CsPb_0.6_Sn_0.4_I_3_ QDs are more uniform than CsPbI_3_ QDs. The higher uniformity of the CsPb_0.6_Sn_0.4_I_3_ QDs resulted in a better‐aligned arrangement compared to CsPbI_3_. Scanning electron microscopy (SEM) showed a more aligned morphology in CsPb_0.6_Sn_0.4_I_3_ films (Figure 1e,h), suggesting reduced lattice distortion resulting from partial Pb–Sn substitution.^[^ ^36^ ^]^ As corroborated by TEM, the smaller ionic radius of Sn decreases the octahedral tilt in the perovskite lattice, thereby mitigating the formation of the orthorhombic non‐perovskite δ‐phase and stabilizing the cubic phase.

Grazing incidence wide‐angle X‐ray scattering (GIWAXS) was performed on PQD‐coated silicon substrates to investigate the crystallinity (Figure 1f,i). In contrast to the ring‐shaped diffraction peaks observed for CsPbI_3_, CsPb_0.6_Sn_0.4_I_3_ exhibited dot‐shaped peaks, indicative of enhanced crystal alignment. Azimuthal integration of the α‐phase (100) plane at q_xy_ ≈0.93 Å^−1^ (red circles, Figure 1j) showed a stronger peak intensity at 90° for CsPb_0.6_Sn_0.4_I_3_, with reduced intensity at other angles, reflecting superior orientation of the crystallites. Similarly, although the (110) phase at q_xy_ ≈1.3 Å^−1^ (white circles, Figure 1k) showed a lower baseline intensity for CsPb_0.6_Sn_0.4_I_3_ compared with CsPbI_3_, the peak intensities were comparable, further corroborating the transition from ring‐like to dot‐like GIWAXS patterns.

X‐ray diffraction (XRD) confirmed the presence of a well‐aligned α‐phase in both PQDs, exhibiting pronounced peaks around 14° and 28° (Figure S4, Supporting Information). The absence of additional peaks indicates minimal impurities or secondary phases. Although the XRD peaks of CsPb_0.6_Sn_0.4_I_3_ were slightly shifted by Sn incorporation, the shift remained difficult to distinguish due to the similarity between alloyed and pure phases.

The absorbance spectra of PQD thin films on glass substrates show an extended absorption range compared to QDs in solution state, with CsPb_0.6_Sn_0.4_I_3_ up to ≈1000 nm (Figure S5a, Supporting Information). This enhancement is attributed to solid‐state effects such as improved light trapping and film morphology. Reflectance measurements on glass also show reduced reflectance beyond the main absorption edge (Figure S5c, Supporting Information), further supporting the improved long‐wavelength absorption in thin films. When coated on silicon, CsPb_0.6_Sn_0.4_I_3_ exhibits lower reflectance in the SWIR region than CsPbI_3_, indicating enhanced infrared absorption. Absorption (A) was calculated as A = 1−R−T where R is reflectance and T is transmittance, but because the silicon substrate and metal electrode block transmission, absorption can be approximated as A ≈ 1−R, highlighting the superior SWIR performance of CsPb_0.6_Sn_0.4_I_3_ films.

To construct the band diagrams, ultraviolet photoelectron spectroscopy (UPS) was performed on PQD‐coated silicon substrates (Figure S6a,b, Supporting Information). The work functions (*WF*) and energy levels of valence band (*E*_VB_) were calculated using the equations

(2) $$WF=h\upsilon -E_{\text{cutoff}}$$

(3) $$E_{\text{VB}}=-\left(WF+E_{\text{initial}}\right)$$

where the photon energy (hυ) is 21.2 eV, *E*_cutoff_ is the cut‐off energy at high binding energy, and *E*_initial_ is the onset energy at low binding energy. The *E*_VB_ were approximately −5.14 eV for CsPbI_3_ and −5.41 eV for CsPb_0.6_Sn_0.4_I_3_. The addition of the respective bandgaps yielded conduction band energies (E_CB_) of approximately −3.32 eV for CsPbI_3_ and −3.91 eV for CsPb_0.6_Sn_0.4_I_3`_. These values are depicted in the schematic band diagram (Figure 1L).

**Figure 2:**
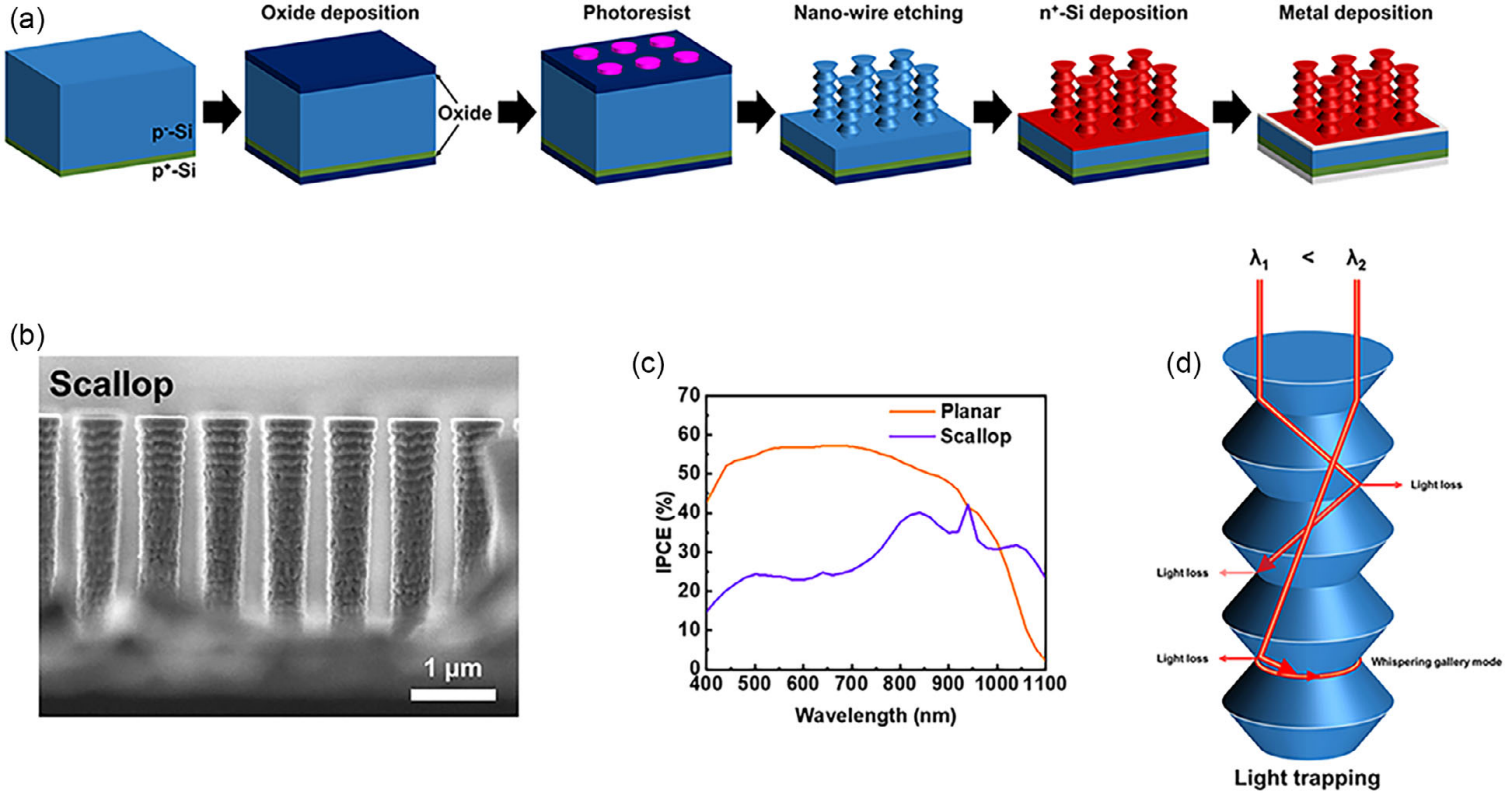
a) Fabrication process of silicon nanowire (scallop) photodetector. b) Cross‐sectional SEM of scallop photodetector. c) Incident photon to current conversion efficiency (IPCE) of planar and scallop photodetector. d) Scheme of the light rapping in scallop structure.

To improve SWIR response of silicon, which is inherently weak beyond ≈1000 nm, a scallop‐shaped nanowire structure was devised. The fabrication workflow is illustrated in **Figure** **2**a. Briefly, a p‐type silicon wafer was coated with an oxide layer, followed by lithographically defined nanodots and subsequent etching to form the scallop‐like nanowire array. An n‐type silicon layer was then deposited atop this scalloped interface to establish a p–n junction. Detailed fabrication procedures are provided in the Experimental Methods section. Cross‐sectional SEM images confirmed the formation of the scallop‐shaped nanowire morphology (Figure 2b).

Figure 2c compares the incident photon‐to‐current conversion efficiency (IPCE) of planar versus scallop‐structured devices spanning the visible to near‐infrared range (400–1000 nm) and extending into the SWIR (>1000 nm). Although planar structures exhibited higher IPCE in the 400–1000 nm window, the scallop configuration delivered superior performance at wavelengths above 1000 nm. This improvement is attributed to whispering‐gallery modes generated within the concave scallop structures.^[^ ^16^ ^]^ Long‐wavelength photons partially refract and repeatedly reflect from the scallop walls, increasing their optical path length and boosting SWIR absorption and subsequent charge carrier generation. Furthermore, any light escaping one scallop can be reabsorbed by adjacent structures, thereby enhancing overall device efficiency (Figure 2d).

**Figure 3:**
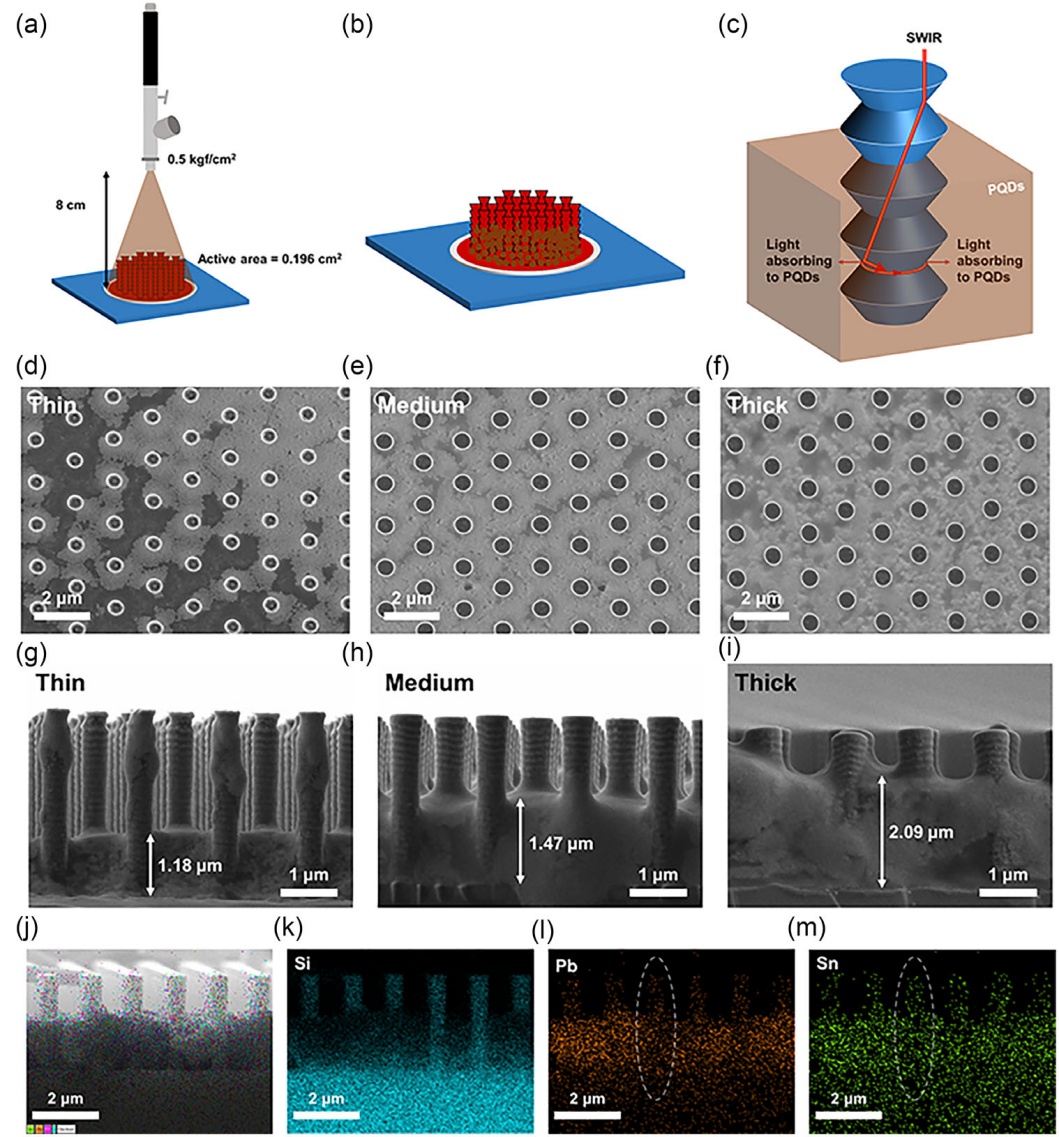
a) Spray coating method of PQDs on silicon scallop photodetector and b) scheme of the PQDs on scallop photodetector. c) Light trapping and absorbance effect of the PQDs coated scallop photodetector. d–f) Top‐view SEM of the device according to PQDs thickness and g–i) cross‐sectional SEM (thin, 2 cycles; medium, 4 cycles; and thick, 6 cycles). j–m) EDS images of CsPb_0.6_Sn_0.4_I_3_ on scallop structure.

To construct the tandem photodetector, PQDs were applied to the scallop‐shaped silicon device via a spray‐coating process (**Figure** **3**a). This procedure was carried out at a pressure of 5 kgf cm^−2^ and a substrate‐to‐nozzle distance of 8 cm. By varying the number of spray cycles (Figure 3b), the thickness of the PQD film was precisely tuned. Complete details regarding the spray‐coating protocol are provided in the Experimental Methods section.

The PQD film deposited on the scallop structure enhances device performance through three principal mechanisms (Figure 3c): (i) filling the interstitial regions within the scallop topography to enable SWIR absorption in areas that would otherwise be poorly responsive, (ii) reabsorbing whispering‐gallery mode‐leaked photons and converting them into additional charge carriers, thereby boosting both the IPCE and photocurrent, and (iii) reducing reflectance, providing an opportunity for silicon to absorb long‐wavelength light. In conventional scallop‐based silicon devices, most light absorption is confined to the top region of the scallop, leaving the lower portions underutilized. Integrating CsPb_0.6_Sn_0.4_I_3_ PQDs, optimized for SWIR absorption, into these deeper scallop regions substantially increases overall absorption. Furthermore, photons not absorbed in the top portion are reabsorbed by the PQDs, yielding extra charge carriers, while the PQD layer also facilitates efficient charge transfer into the silicon due to the favorable energy level alignment, as confirmed by UPS. In the long‐wavelength region, PQD layer acts as an anti‐reflection and light‐scattering coating, which provides a longer effective absorption path length. This extended absorption path length enables silicon to absorb longer wavelength light.^[^ ^37^ ^]^ Subsequent IPCE, current–voltage (I–V), and response time evaluations corroborated these enhancements.

Top‐view SEM images reveal the distribution of PQDs (Figure 3d–f), which varies according to the number of spray cycles. Cross‐sectional SEM analyses confirm the incremental growth of the PQD layer (Figure 3g–i), ranging from 1.18 μm for the thinnest film (2 cycles) to 1.47 μm (medium; 4 cycles) and 2.09 μm (thick; 6 cycles), demonstrating controllable layer thickness via spray coating. Energy dispersive X‐ray spectroscopy (EDS) mapping shows that silicon remains uniformly present in the nanowire scaffold (Figure 3j–m), while lead and tin are localized predominantly within the PQD film and nanowire sidewall. Although the Pb and Sn signals diminish slightly toward the apex of the nanowire structure, these results indicate effective PQD deposition across both the substrate base and the vertical nanowire pillars. Furthermore, EDS analyses confirm a Pb:Sn atomic ratio of ≈6:4, in agreement with XPS measurements.

The IPCE of the photodetectors was measured under zero bias across a wavelength range of 350–1100 nm. IPCE, which quantifies the ratio of collected charge carriers to incident photons, is a key metric for assessing photodetector efficiency. To investigate the impact of PQD incorporation, PQDs of varying thickness were deposited onto scallop‐structured silicon.

**Figure 4:**
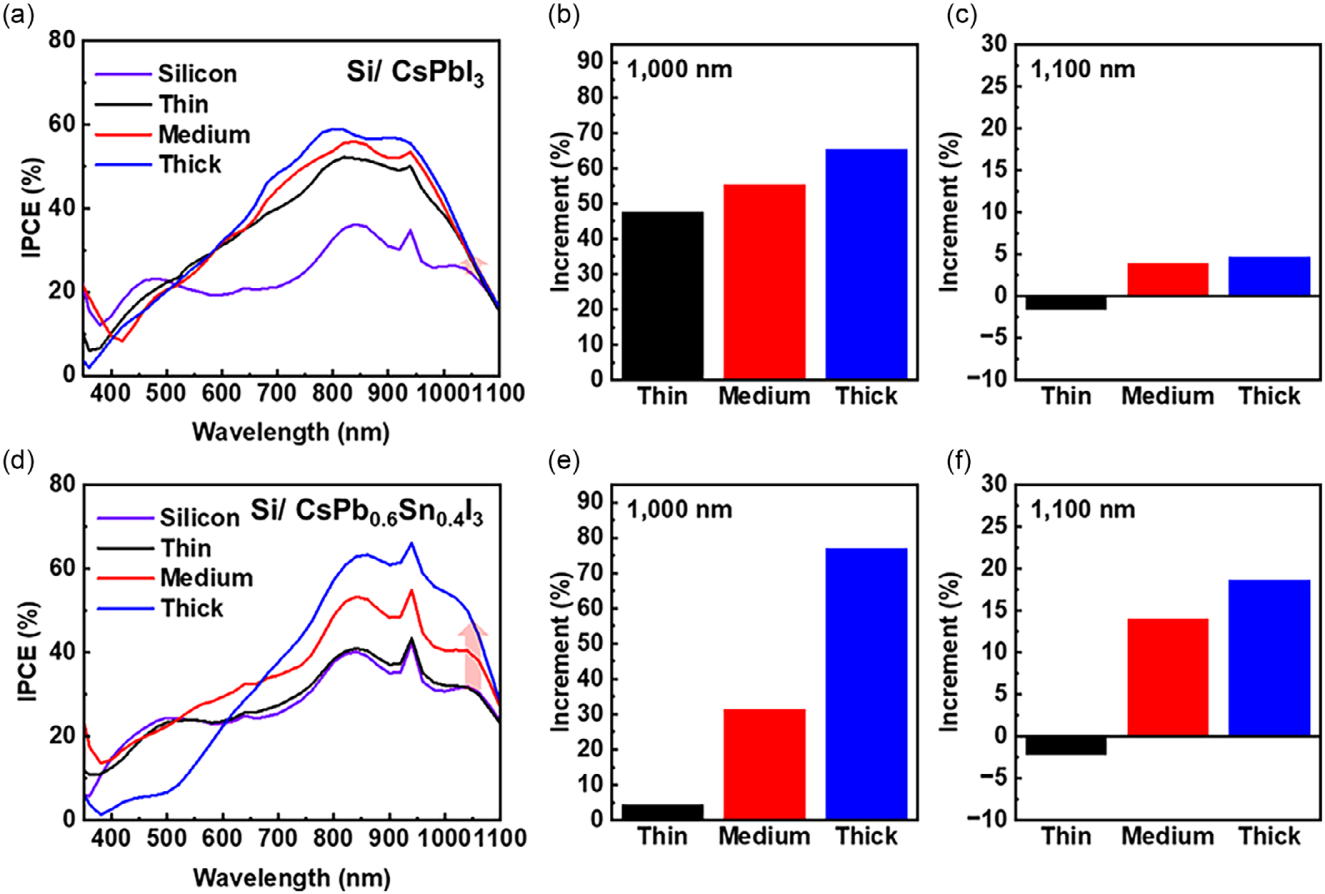
a) IPCE of the Si/CsPbI_3_ and b,c) increment at 1000 nm and 1100 nm. d) IPCE of the Si/CsPb_0.6_Sn_0.4_I_3_ and e,f) increment at 1000 nm and 1100 nm.

For the Si/CsPbI_3_ devices, the IPCE increased proportionally with PQD thickness, attaining a maximum of 58.9% at 800 nm (**Figure** **4**a). In contrast, the Si/CsPb_0.6_Sn_0.4_I_3_ devices displayed a decline in IPCE at shorter wavelengths as the PQD layer thickened, yet a marked enhancement was observed at longer wavelengths, particularly in the SWIR range. The highest IPCE measured for Si/CsPb_0.6_Sn_0.4_I_3_ was 63.3% at 860 nm (Figure 4d).

A comparative analysis of the IPCE in the SWIR regime further highlights the influence of tin substitution. At 1000 nm, Si/CsPbI_3_ samples exhibited incremental IPCE enhancements of 47.8%, 55.6%, and 65.6% relative to bare silicon (Figure 4b). Notably, the Si/CsPb_0.6_Sn_0.4_I_3_ counterparts showed more pronounced improvements of 4.4%, 31.5%, and 77.1%, respectively, with increasing PQD thickness (Figure 4e). Beyond 1000 nm, the performance disparity between CsPbI_3_ and CsPb_0.6_Sn_0.4_I_3_ became even more apparent: at 1100 nm, Si/CsPbI_3_ exhibited negligible gains (<5%) over bare silicon, whereas Si/CsPb_0.6_Sn_0.4_I_3_ achieved substantial improvements of 14.0% (medium thickness) and 18.7% (thick layer) (Figure 4c,f).

These findings underscore the effectiveness of incorporating PQDs—particularly CsPb_0.6_Sn_0.4_I_3_—in enhancing SWIR responsivity. Increasing the PQD film thickness consistently boosted the photodetector's IPCE, demonstrating that a tailored PQD architecture can significantly improve performance at the operational wavelength range of silicon‐based devices.

**Figure 5:**
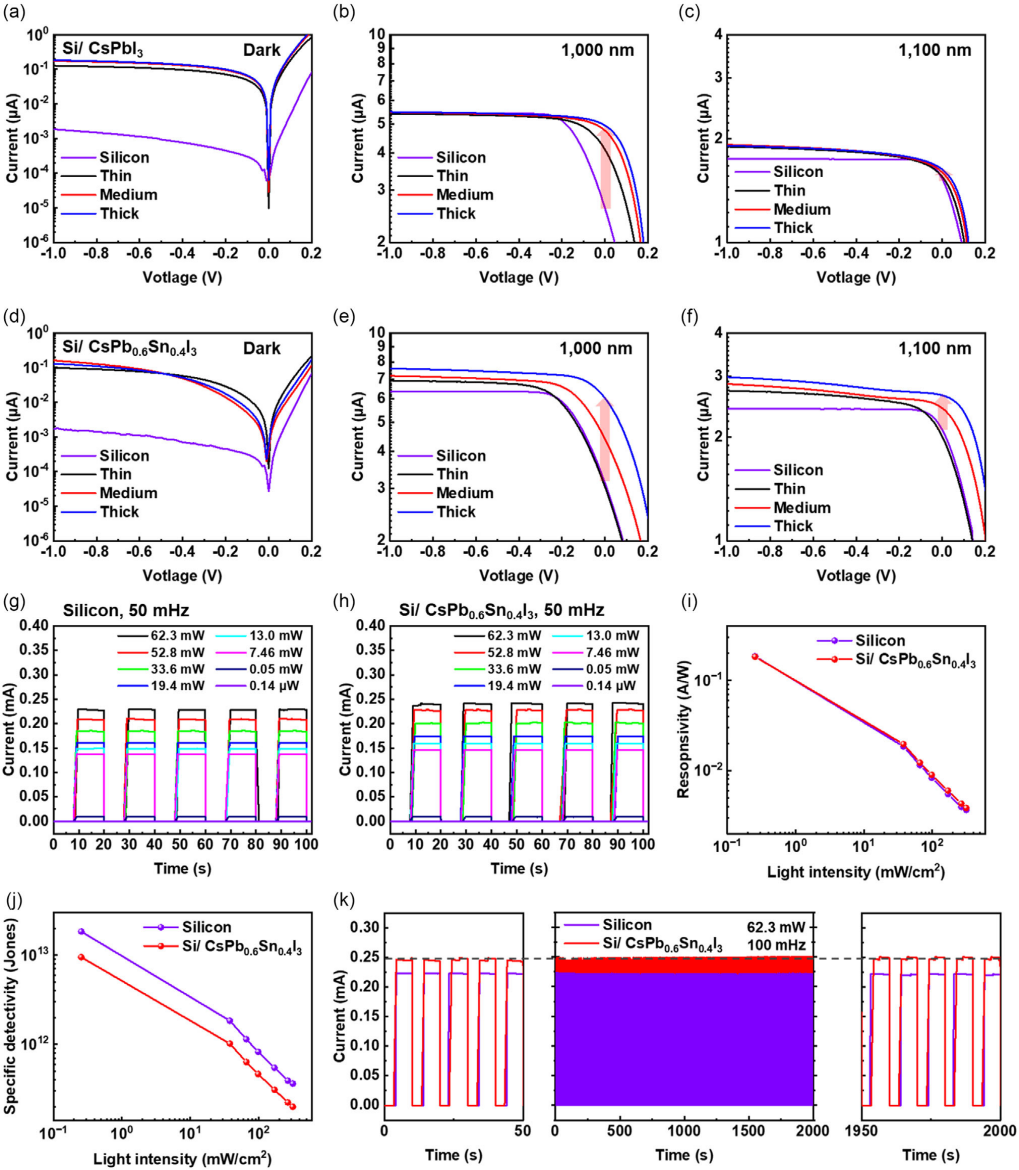
a–c) Current–voltage curve of Si/CsPbI_3_ at dark, 1000 nm and 1100 nm. d–f) Current–voltage curve of Si/CsPb_0.6_Sn_0.4_I_3_ at dark, 1000 nm and 1100 nm. On/off current of g) the silicon device and h) Si/CsPb_0.6_Sn_0.4_I_3_ under 1064 nm light at 50 mHz. i) Responsivity and j) specific detectivity of each device. k) 200‐cycle on/off ratio of silicon device and Si/CsPb_0.6_Sn_0.4_I_3_ at 100 mHz in ambient air (20–23 °C and RH 50–60%) without any encapsulation.

To evaluate the SWIR‐region performance, the dark current and photocurrent were measured under illumination at wavelengths of 1000 nm and 1100 nm. At an applied bias of −1 V, Si/CsPbI_3_ devices exhibited a relatively stable dark current of ≈1.8 μA for both medium‐ and thick‐PQD layers (**Figure** **5**a). By contrast, Si/CsPb_0.6_Sn_0.4_I_3_ devices attained lower dark currents under the same bias, registering 0.1 μA (thin), 0.16 μA (medium), and 0.13 μA (thick) (Figure 5d). Due to the higher conduction band of the CsPbI_3_ and CsPb_0.6_Sn_0.4_I_3_ compared to n‐type silicon, electrons flow from the PQDs to the silicon, generating dark current. Dark current of the CsPb_0.6_Sn_0.4_I_3_ device was lower than that of the CsPbI_3_ device, because traps generated by oxidized Sn^4+^ capture electrons.

Under 1000 nm illumination, Si/CsPbI_3_ displayed minimal improvement over bare silicon, with the photocurrent largely independent of PQD‐layer thickness (Figure 5b). Conversely, Si/CsPb_0.6_Sn_0.4_I_3_ showed a progressive photocurrent increase with increasing PQD thickness: 6.91 μA (thin), 7.17 μA (medium), and 7.59 μA (thick), compared to 6.35 μA for bare silicon (Figure 5e). At 1100 nm, the Si/CsPbI_3_ device revealed only marginal enhancements (<0.02 μA difference relative to bare silicon), whereas Si/CsPb_0.6_Sn_0.4_I_3_ demonstrated gains of 2.73 μA (thin), 2.85 μA (medium), and 2.98 μA (thick) (Figure 5c,f). These findings emphasize the beneficial effect of tin‐substituted PQDs on SWIR photocurrent generation.

The photodetector's responsivity (R) and specific detectivity (D*) were determined through on/off current measurements under 1064 nm illumination (62.3 mW) at varying light intensities and a frequency of 50 mHz (Figure 5g,h). Responsivity was calculated using the following relationship^[^ ^38^ ^]^

(4) $$R=\frac{I_{l}-I_{d}}{P_{i}}$$

where *I*_l_ and *I*_d_ denote illuminated and dark currents, respectively, and *P*_i_ is the incident optical power. As illustrated in Figure 5i, the Si/CsPb_0.6_Sn_0.4_I_3_ devices exhibited consistently higher responsivity compared with bare silicon. Although CsPbI_3_‐based devices did not demonstrate notable photocurrent enhancements (Figure S7a,b, Supporting Information), slight improvements in responsivity were observed under varying light intensities (Figure S7c, Supporting Information).

Specific detectivity (D*) was derived from on/off current measurements using the following equations^[^ ^39^, ^40^ ^]^

(5) $$D^{*}=\frac{\sqrt{A\Delta f}}{NEP}$$

(6) $$\Delta f=\frac{1}{2t_{c}}$$

(7) $$\text{NEP}=\frac{\sqrt{2qI_{d}\Delta f}}{R}$$

where *A* is the active area, Δf is the detection bandwidth, *t*_c_ is the time constant determined as the time required to reach 63.2% of the final photocurrent, and *q* is the electronic charge. The time constant was determined from the rise‐time analysis at 25 Hz (Figure S8, Supporting Information). For Si/CsPb_0.6_Sn_0.4_I_3_, D* was slightly lower than that of bare silicon (Figure 5j), whereas CsPbI_3_‐based detectors showed no discernible variation in detectivity (Figure S7d, Supporting Information).

Device stability was assessed by performing on/off current measurements for 200 cycles at a power of 62.3 mW and a frequency of 100 mHz in ambient air (20–23 °C and RH 50–60%) without any encapsulation. Figure 5k confirms stable photocurrent in Si/CsPb_0.6_Sn_0.4_I_3_ devices, while CsPbI_3_‐based detectors demonstrated photocurrent degradation over repeated cycles (Figure S7e, Supporting Information), likely reflecting the lower structural stability of CsPbI_3_. Partial Pb–Sn substitution reduces lattice distortion in CsPb_0.6_Sn_0.4_I_3_, thus enhancing structural resilience.

Transient response characteristics, including rise and decay times, were examined under 1064 nm illumination at 25 Hz. The decay times for Si/CsPbI_3_ and Si/CsPb_0.6_Sn_0.4_I_3_ were measured to be 5.749 ms and 6.130 ms, respectively—both faster than that of bare silicon (9.4 ms) (Figure S8a, Supporting Information). Similarly, the rise times of Si/CsPbI_3_ (7.173 μs) and Si/CsPb_0.6_Sn_0.4_I_3_ (8.270 μs) were shorter than that of bare silicon (8.661 μs), demonstrating that PQD incorporation significantly enhances photodetector response speed (Figure S8b, Supporting Information). This improvement is maintained across different PQD thicknesses, as the nanowire‐based geometry facilitates efficient charge collection along the scallop sidewalls, mitigating the typical trade‐off between increased absorption and slower response.

**Table** **1** summarizes the performance of several representative SWIR photodetectors. Our PQD‐functionalized silicon photodetector achieves a responsivity of 0.36 A W^−1^ at 1064 nm and a detectivity of 9.48 × 10^12^ Jones, offering a cost‐effective alternative to conventional III–V detectors such as InGaAs. While the responsivity is currently lower than that of state‐of‐the‐art performance from PbS QD hybrid devices, the use of reduced‐lead perovskite QDs offers improved environmental compatibility. Moreover, the solution‐based spray‐coating fabrication method is readily applicable to large‐area, low‐cost manufacturing.

**Table 1:** Material and photodetection performance of SWIR photodetectors.

**Material**	**Wavelength [nm]**	**Responsivity [A/W]**	**Specific detectivity [Jones]**	**Rise/decay time**	**Bias [V]**	**Year**	**Ref.**
Our research	1064	0.18	9.48 × 10^12^	8.27 μs 6.13 ms	0	–	–
Flexible InGaAs/InP	1550	0.52	5.18 × 10^11^	116 ns 951 ns	−0.1	2022	[6]
CsPbBr_3_/Silicon	900	0.304	1.17 × 10^13^	75 ms 82 ms	0	2024	[37]
CdZnS/ZnS /Silicon	900	0.391	1.37 × 10^13^	75 ms 137 ms	0	2024	
ITO/PbS‐TBAI/SiN_x_/Si	1064	0.68	7.74 × 10^10^	0.16 ms 0.32 ms	0.05	2022	[43]
InGaAs	1550	0.48	–	–	0	2023	[44]
GaAsSb/GaAs	900	100	1.10 × 10^14^	–	−1	2020	[45]
CuInSe_2_	1100	0.015	2.50 × 10^11^	– 1.9 μs	0	2021	[46]
Mn‐doped CuInSe_2_	1100	0.03	4.20 × 10^12^	– 0.76 μs	0	2021	
PbS/CuSCN	1200	0.003	7.00 × 10^10^	50 μs 110 μs	−1	2020	[47]
2D GaGeTe	1310	57	2.80 × 10^7^	0.53 s 0.66 s	2	2021	[48]
Quinoid terminal group	1160	0.13	1.68 × 10^12^	35.8 μs 36 μs	0	2024	[49]
InAs/GaSb/AlSb/GaSb	1700	0.54	3.00 × 10^13^	–	−2	2021	[50]
PbS CNT TFT	1300	1.65 × 10^4^	5.60 × 10^13^	0.57 ms 0.97 ms	−0.1	2022	[51]
InSb QD	1370	0.126	3.90 × 10^12^	–	0	2024	[52]
*p*‐PbS/WS_2_	1550	0.179	4.11 × 10^11^	–	0	2023	[53]
α‐In_2_Se_3_	1550	0.16	5.61 × 10^9^	8 ms 10 ms	0	2019	[54]
PTB7‐Th:CS‐1	1300	0.016	2.96 × 10^10^	5.8 μs 7.4 μs	0	2025	[55]

To further improve performance, interface engineering strategies such as defect passivation, surface ligand optimization, and energy band alignment tuning will be explored to enhance charge extraction across the QD/Si heterojunction. Despite these challenges, the proposed Si/CsPb_0.6_Sn_0.4_I_3_ device already demonstrates enhanced SWIR responsivity and fast temporal response (rise time: 8.27 μs, decay time: 6.13 ms), comparable to established high‐performance materials (e.g., Mn‐doped CuInSe_2_). Despite the material instability of lead‐tin mixed perovskites, these results underscore the potential of PQD‐engineered silicon architectures, when combined with appropriate encapsulation techniques, for next‐generation SWIR sensing in autonomous systems and industrial applications.

## 3 Conclusion

In this study, we introduced a high‐performance silicon‐based infrared photodetector by combining a scallop‐structured silicon nanowire array with tin‐substituted PQDs. This integrated architecture effectively overcomes the intrinsic absorption limitations of silicon, enabling enhanced sensitivity in the SWIR regime beyond 1000 nm. The scallop nanostructure confines and guides infrared light, while the incorporated PQDs facilitate efficient charge transfer, yielding marked improvements in responsivity and overall detection capability. Notably, the optimized CsPb_0.6_Sn_0.4_I_3_ PQDs achieved superior photodetection at 1000 nm and 1100 nm, accompanied by a response time of ≈6 ms—faster than conventional silicon photodetectors—and sustained stable operation across multiple measurement cycles.

These findings present a scalable, cost‐effective pathway to broaden the operational wavelength range of silicon photodetectors, providing an economical alternative to InGaAs‐based devices. The demonstrated infrared detection capabilities hold significant promise for next‐generation autonomous vision systems, biomedical diagnostics, and industrial sensing applications. Future research may focus on further refining the PQD composition and nanostructural design to further optimize device performance and long‐term stability.

## 4 Experimental Section

4.1

4.1.1

##### Materials

Cesium carbonate (Cs_2_CO_3_; trace metals basis, 99.995%), 1‐octadecene (ODE; technical grade, 90%), oleic acid (OA; technical grade, 90%), oleylamine (OAm; technical grade, 70%), trioctylphosphine (TOP; 97%), methyl acetate (MeOAc; anhydrous, 99.5%), hexane (anhydrous, 95%), and octane (anhydrous, ≥99%) were purchased from Sigma‐Aldrich. PbI_2_ (99.99%, trace metals basis) was purchased from TCI. Tin iodide (SnI_2_; ultra dry, 99.999%, metal basis) was purchased from thermo scientific.

##### PQDs Synthesis

*CsPbI*_*3*_: The CsPbI_3_ QDs were synthesized using modified hot‐injection methods.^[^ ^41^, ^42^ ^]^ In a 100 mL three‐neck flask, Cs_2_CO_3_ (0.204 g) and OA (0.625 mL) were mixed in 10 mL of ODE. The mixture was heated at 120 °C for 1 h under vacuum with vigorous stirring and then heated at 150 °C under nitrogen until all the Cs_2_CO_3_ was dissolved. Prepared solution (Cs‐oleate) was stored in N_2_ glove box and annealed at 110 °C prior to use. In a 100 mL three‐neck flask, PbI_2_ (0.50 g; 1.08 mmol), OA (2.5 mL), and OAm (2.5 mL) were mixed in 25 mL of ODE. The mixture was stirred vigorously at 120 °C for 1 h under vacuum. Under N_2_ atmosphere, the mixture was heated to 150 °C and then injected 2 mL of Cs‐oleate. Five seconds after injection, the three‐neck flask was quenched into an ice bath. Crude solution was purified by adding 60 mL of MeOAc and centrifuged at 5000 rpm for 3 min. The supernatant was discarded, and the precipitate was dispersed in 10 mL of hexane. The dispersion solution was purified by adding 14 mL followed by centrifugation at 5000 rpm for 3 min. After discarding the supernatant, the precipitate was dispersed in 15 mL hexane. The dispersion was centrifuged repeatedly until the precipitate disappeared. Prepared PQDs dispersion was stored in refrigerator overnight and centrifuged at 5000 rpm for 3 min before use. To use at device, the hexane was removed from PQDs solution and dispersed in octane at 50 mg/mL.

*CsPb*_*0.6*_ *Sn*_*0.4*_ *I*_*3*_: In a N_2_ glove box, SnI_2_ (1.4901 g; 4 mmol) and PbI_2_ (0.4610 g; 1 mmol) were stirred in 5 mL of TOP at 90 °C for 5 h. Prior to use, the precursor was centrifuged at 4000 rpm for 3 min to remove residues. In a three‐neck flask, Cs_2_CO_3_ (0.12 g), OA (0.4 mL), and OAm (0.4 mL) were mixed in 16 mL of ODE. The mixture was dissolved at 100 °C for 3 h under a vacuum atmosphere. After then, the mixture was heated at 120 °C under N_2_ until the mixture became transparent. The mixture was heated at 160 °C and injected 2.5 mL of TOP‐(PbI_2_&SnI_2_) solution. Five seconds after injection, the three‐neck flask was quenched into an ice bath. To purify the PQDs solution, 25 mL MeOAc was added in crude solution and centrifuged at 4000 rpm for 3 min. The precipitate was dispersed in 5 mL hexane. The dispersion was stored in refrigerator for overnight and centrifuged at 4000 rpm for 3 min before use. To use at device, the hexane was removed from PQDs solution and dispersed in octane at 50 mg mL^−1^.

##### Device Fabrication

*Silicon photodetector*: A clean p‐type silicon was prepared as a substrate. An oxide mask was used to fabricate the scallop‐shaped nanostructure. The silicon oxide layer was deposited using LPCVD. Nanodot patterns using photoresist were formed on the oxide mask, which facilitates the formation of scallop‐shaped nanowire during the etch process. All the nanowires had same height (3 μm), gap between nanowires (1.5 μm) and diameter of pillars (600 nm). Active area is 0.196 cm^2^. To construct the scallop‐shaped photodetector, n‐type silicon layer with high doping concentration was deposited epitaxially. The photodetector was rinsed with acetone and IPA. The silicon substrate was etched to form an area to deposit metal, and aluminum was deposited in the etch region.

*Tandem photodetector‐Spray coating*: Connect the spray gun to nitrogen gas and spray coat the PQDs (50 mg mL^−1^ in octane) on Si‐wafer at a pressure of 0.5 kgf cm^2^. Distance between spray gun and Si‐wafer is about 8 cm. PQDs were sprayed twice for thin, 4 times for medium and 6 times for thick to analyze properties according to coating thickness.

##### Characterization

PL data were obtained using FluoroMax (HORIBA), and UV‐vis absorbance data were obtained using OPTIZEN POP (KLAB). TEM images of the PQDs were obtained using field‐emission TEM (JEM‐ARM200F, JEOL) at the Center for University‐wide Research Facilities (CURF) of Jeonbuk National University. SEM images of the PQDs were obtained using field‐emission SEM (S‐4700, Hitachi) at the Center for University wide Research Facilities (CURF) of Jeonbuk National University. To confirm the ordered arrangement of PQDs, SEM samples were prepared by spray coating 4 times on silicon wafer, followed by sputtering of Pt for high resolution SEM. EDS data were obtained using an Aztec Energy instrument (Oxford Instruments) at the CURF. GIWAXS measurements were conducted at the 3C and 9 A beamlines at the Pohang Accelerator Laboratory (PAL), Republic of Korea. XPS measurements were conducted using a Nexsa XPS system (Thermo Fisher Scientific) equipped with Al K α (1486.6 eV) as the X‐ray source at the Jeonju Center of the Korea Basic Science Institute (KBSI). UPS was measured using a Nexsa XPS system (Thermo Fisher Scientific) equipped with He I (21.2 eV) as the UV source. XRD was measured using a D8 ADVANCE (Bruker) equipped with Cu X‐ray tube generator at the Jeonbuk National University Research Facilities Center. IPCE was measured using monochrometer in dark shield box (MSTECH). Spectral responsivity and specific detectivity were measured using 1,064 nm laser (Thorlabs) in dark shield box. On/off ratio data were measured using 1064 nm laser and oscilloscope (Tektronix) in dark shield box.

## Conflict of Interest

The authors declare no conflict of interest.

## Supporting information

Supplementary Material

## Data Availability

The data that support the findings of this study are available from the corresponding author upon reasonable request.

## References

[1] F. Cao , L. Liu , L. Li , Mater. Today 2023, 62, 327.

[2] Z. Guo , J. Wang , J. Du , D. Wu , L. Zeng , Y. H. Tsang , D. Wu , Y. Wang , Y. Ding , P. Lin , Nano Energy 2025, 133, 110452.

[3] P. Y. Huang , Y. Y. Zhang , P. C. Tsai , R. J. Chung , Y. T. Tsai , M. K. Leung , S. Y. Lin , M. H. Fang , Adv. Opt. Mater. 2024, 12, 2302062.

[4] W. Kim , Y. Seo , D. Ahn , I. S. Kim , C. Balamurugan , G. Y. Jung , S. Kwon , H. Kim , Y. Pak , Adv. Sci. 2024, 11, 2308840. 10.1002/advs.202308840 PMC11151070 38460159

[5] A. K. Katiyar , K. Y. Thai , W. S. Yun , J. Lee , J.‐H. Ahn , Sci. Adv. 2020, 6, eabb0576. 32832687 10.1126/sciadv.abb0576 PMC7439440

[6] X. Li , J. Zhang , C. Yue , X. Tang , Z. Gao , Y. Jiang , C. Du , Z. Deng , H. Jia , W. Wang , Sci. Rep. 2022, 12, 7681. 35538226 10.1038/s41598-022-11946-7 PMC9090829

[7] J. Klem , J. Kim , M. Cich , G. Keeler , S. Hawkins , T. Fortune , Appl. Phys. Lett. 2009, 95.

[8] N. Işık , S. Kocaman , Infrared Phys. Technol. 2024, 143, 105590.

[9] Y.‐C. Kao , H.‐M. Chou , S.‐C. Hsu , A. Lin , C.‐C. Lin , Z.‐H. Shih , C.‐L. Chang , H.‐F. Hong , R.‐H. Horng , Sci. Rep. 2019, 9, 4308. 30867491 10.1038/s41598-019-40727-y PMC6416321

[10] R. Beeler , J. Mathews , J. Tolle , R. Roucka , A. Chizmeshya , R. Juday , S. Bagchi , J. Menendez , J. Kouvetakis , Sol.Energy Mater. Sol. Cells 2010, 94, 2362.

[11] L. Wang , Y. Pan , J.‐L. Xing , J.‐B. Mao , Y.‐J. Ba , L. Cao , M.‐L. Wang , C.‐Y. Wu , X. Zhang , L.‐B. Luo , IEEE Electron Device Lett. 2023, 44, 1148.

[12] Y. Dai , X. Wang , W. Peng , C. Xu , C. Wu , K. Dong , R. Liu , Z. L. Wang , Adv.Mater 2018, 30, 1705893. 10.1002/adma.201705893 29334148

[13] J. Wu , M. Wei , J. Mu , H. Ma , C. Zhong , Y. Ye , C. Sun , B. Tang , L. Wang , J. Li , ACS nano 2021, 15, 15982. 34652907 10.1021/acsnano.1c04359

[14] F. X. Liang , X. Y. Zhao , J. J. Jiang , J. G. Hu , W. Q. Xie , J. Lv , Z. X. Zhang , D. Wu , L. B. Luo , Small 2019, 15, 1903831. 10.1002/smll.201903831 31513340

[15] H. Zhang , B. Man , Q. Zhang , ACS Appl. Mater.Interfaces 2017, 9, 14067. 28398029 10.1021/acsami.7b01098

[16] M. Choi , H.‐T. Kwak , H. Kim , H. Yoo , J. H. Park , C.‐K. Baek , Opt. Express 2023, 31, 38013. 38017919 10.1364/OE.503871

[17] H. Wook Shin , S. Jun Lee , D. Gun Kim , M.‐H. Bae , J. Heo , K. Jin Choi , W. Jun Choi , J.‐W. Choe , J. Cheol Shin,, Sci. Rep. 2015, 5, 10764. 26035286 10.1038/srep10764 PMC4451803

[18] A. Sharma , S. Zaidi , P. Logofatu , S. Brueck , IEEE J. Quantum Electron. 2002, 38, 1651.

[19] J.‐H. Yun , J. Kim , Y. C. Park , J. Appl. Phys. 2014, 116.

[20] Z. Zhang , W. Gan , J. Li , Z. Kong , Y. Han , Y. Liu , G. Wang , Z. Wu , J. Yu , Q. Zhang , Mater. Sci. Semicond. Process. 2022, 140, 106337.

[21] W. Xu , H. Yin , X. Ma , P. Hong , M. Xu , L. Meng , Nanoscale Res. Lett. 2015, 10, 1. 26055484 10.1186/s11671-015-0958-4 PMC4456591

[22] A. Martinez , K. Kalna , J. Barker , A. Asenov , Phys. E: Low‐dimensional Systems and Nanostructures 2007, 37, 168.

[23] S. Sato , W. Li , K. Kakushima , K. Ohmori , K. Natori , K. Yamada , H. Iwai , Appl. Phys. Lett. 2011, 98.

[24] W. Liao , W. Qian , J. An , L. Liang , Z. Hu , J. Wang , L. Yu , Nano‐Micro Lett. 2025, 17, 154. 10.1007/s40820-025-01674-8 PMC11839962 39969658

[25] V. Yeddu , G. Seo , M. Bamidele , H. Jung , H. Yu , J. W. Lee , J. Lee , D. Y. Kim , ACS Appl. Electron. Mater. 2022, 4, 1206.

[26] W. Wang , D. Zhao , F. Zhang , L. Li , M. Du , C. Wang , Y. Yu , Q. Huang , M. Zhang , L. Li , Adv. Funct. Mater. 2017, 27, 1703953.

[27] X. Xu , C. C. Chueh , P. Jing , Z. Yang , X. Shi , T. Zhao , L. Y. Lin , A. K. Y. Jen , Adv. Funct. Mater. 2017, 27, 1701053.

[28] G. Schileo , G. Grancini , J. Mater. Chem. C 2021, 9, 67.

[29] S. Yang , W. Fu , Z. Zhang , H. Chen , C.‐Z. Li , J. Mater. Chem. A 2017, 5, 11462.

[30] E. W.‐G. Diau , E. Jokar , M. Rameez , ACS Energy Lett. 2019, 4, 1930.

[31] B. Li , B. Chang , L. Pan , Z. Li , L. Fu , Z. He , L. Yin , ACS Energy Lett. 2020, 5, 3752.

[32] T. Soto‐Montero , S. Kralj , J. S. Gómez , J. W. Wolffs , N. Rodkey , A. P. Kentgens , M. Morales‐Masis , Chem. Mater. 2024, 36, 6912. 39070671 10.1021/acs.chemmater.4c00935 PMC11270747

[33] C. Zhang , Q. Wan , L. K. Ono , Y. Liu , W. Zheng , Q. Zhang , M. Liu , L. Kong , L. Li , Y. Qi , ACS Energy Lett. 2021, 6, 3545.

[34] J. Pan , Z. Zhao , F. Fang , L. Wang , G. Wang , C. Liu , J. Chen , J. Xie , J. Sun , K. Wang , Adv. Opt. Mater. 2020, 8, 2001494.

[35] A. Mannu , M. E. Di Pietro , A. Mele , Molecules 2020, 25, 1495. 32218347 10.3390/molecules25071495 PMC7180541

[36] A. Swarnkar , W. J. Mir , A. Nag , ACS Energy Lett. 2018, 3, 286.

[37] K. S. R. Bapathi , M. F. Abdelbar , W. Jevasuwan , Q. Zhang , P. H. Borse , S. Badhulika , N. Fukata , Nano Energy 2024, 128, 109832.

[38] S. M. Park , S. W. Park , H. Jin , D. Baek , J. Cha , W. S. Chae , T. K. Lee , M. Kim , Small 2024, 20, 2404958. 10.1002/smll.202404958 39136205

[39] H. P. Wang , S. Li , X. Liu , Z. Shi , X. Fang , J. H. He , Adv. Mater. 2021, 33, 2003309. 10.1002/adma.202003309 33346383

[40] Q. Wang , Y. Zhang , Z. Wei , Chin. J. Chem. 2023, 41, 958.

[41] H. Choi , H. Kim , J. Lim , B.‐J. Chang , S. Song , Macromol. Res. 2024, 32, 825.

[42] S. B. Yoon , S. Hwang , Y. Kim , B.‐G. Kim , H. B. Na , Korean J. Chem. Eng. 2024, 41, 3345.

[43] J. Wang , J. Chen , Surf. Interfaces 2022, 30, 101945.

[44] H. Liu , J. Wang , D. Guo , K. Shen , B. Chen , J. Wu , Nanomaterials 2023, 13, 2895. 37947739 10.3390/nano13212895 PMC10650572

[45] S. Nalamati , S. Devkota , J. Li , R. Lavelle , B. Huet , D. Snyder , A. Penn , R. Garcia , ACS Appl. Electron. Mater. 2020, 2, 3109.

[46] R. Guo , J. Meng , W. Lin , A. Liu , T. Pullerits , K. Zheng , J. Tian , Chem. Eng. J. 2021, 403, 126452.

[47] I. Ka , L. F. Gerlein , I. M. Asuo , S. Bouzidi , D. M. Gedamu , A. Pignolet , F. Rosei , R. Nechache , S. G. Cloutier , ACS Photonics 2020, 7, 1628.

[48] S. R. Tamalampudi , G. Dushaq , J. E. Villegas , N. S. Rajput , B. Paredes , E. Elamurugu , M. S. Rasras , Opt. Express 2021, 29, 39395. 34809305 10.1364/OE.442845

[49] Y. Zhang , J. Chen , J. Yang , M. Fu , Y. Cao , M. Dong , J. Yu , S. Dong , X. Yang , L. Shao , Adv.Mater 2024, 36, 2406950. 10.1002/adma.202406950 39152933

[50] M. Razeghi , A. Dehzangi , J. Li , Results in Optics 2021, 2, 100054.

[51] S. Zhou , Y. Wang , C. Deng , P. Liu , J. Zhang , N. Wei , Z. Zhang , Appl. Phys. Lett. 2022, 120.

[52] H. Seo , H. J. Eun , A. Y. Lee , H. K. Lee , J. H. Kim , S. W. Kim , Adv. Sci. 2024, 11, 2306439. 10.1002/advs.202306439 PMC10811490 38036427

[53] S. H. Kim , D. Lee , S. Moon , J. H. Choi , D. Kim , J. Kim , S. W. Baek , Adv. Funct. Mater. 2023, 33, 2303778.

[54] B. Tang , L. Hou , M. Sun , F. Lv , J. Liao , W. Ji , Q. Chen , Nanoscale 2019, 11, 12817. 31180398 10.1039/c9nr03077h

[55] J. Cong , Z. H. Huang , S. W. Liu , Z. Luo , F. Z. Liu , Z. Chen , K. M. Lee , Y. C. Huang , C. Yang , Small 2025, 21, 2410418. 10.1002/smll.202410418 39935090

