# Supplementary material for: Perovskite Quantum Dot‐Enhanced Silicon Photodetectors for High‐Performance Infrared Sensing
Source: Small Sci. 2025 Jul 3;5(9):2500170. doi: 10.1002/smsc.202500170 (PMC12412522; doi:10.1002/smsc.202500170)
Supplement: Supplementary file 1 — Supplementary Material [file SMSC-5-2500170-s001.pdf]

Supporting Information

Perovskite Quantum Dot-Enhanced Silicon Photodetectors for High-Performance Infrared Sensing

Dohun Baek, Eunseo Nam, Su Min Park, Jeongbeom Cha, Haedam Jin, Hyeongyu Kim, Jihun Lee, Kihyun Kim*, and Min Kim*

Dohun Baek, Su Min Park, Min Kim
School of Chemical Engineering, Jeonbuk National University, Jeonju, 54896 Republic of Korea

Eunseo Nam, Hyeongyu Kim, Jihun Lee, Kihyun Kim
Division of Electronics and Information Engineering and Future Semiconductor Convergence Technology Research Center, Jeonbuk National University, Jeonju, 54896 Republic of Korea

Jeongbeom Cha, Haedam Jin
Department of Integrated Energy-AI, Jeonbuk National University, Jeonju, 54896 Republic of Korea, Department of Intelligent Semiconductor Engineering, University of Seoul, Seoul, 02504 Republic of Korea

Kihyun Kim
Division of Electronics Engineering, Jeonbuk National University, Jeonju, 54896 Republic of Korea
Email: kihyun.kim@jbnu.ac.kr

Min Kim
Department of Chemical Engineering, University of Seoul, Seoul, 02504 Republic of Korea
Email: min.kim@uos.ac.kr

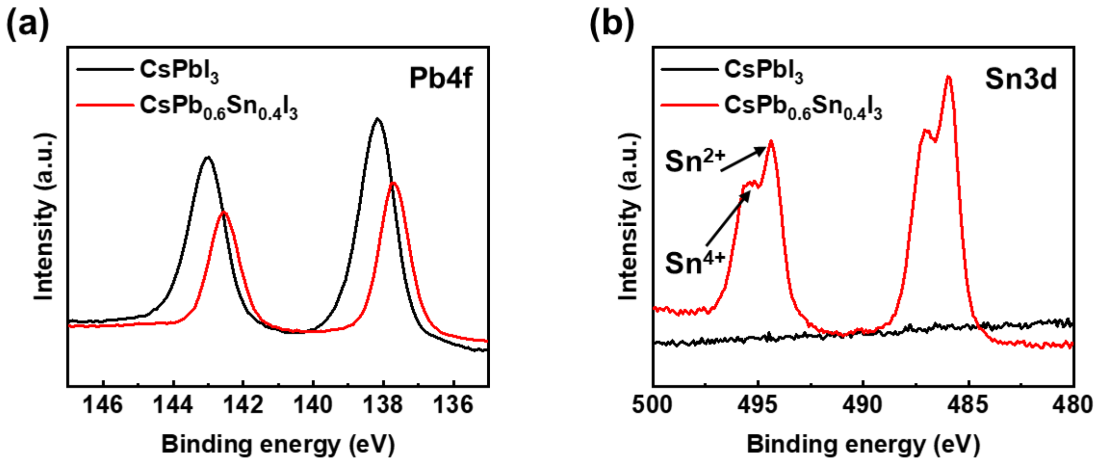

Figure S1. XPS for (a) Pb 4f and (b) Sn 3d of the $CsPbI_3$ and $CsPb_{0.6}Sn_{0.4}I_3$ PQDs.

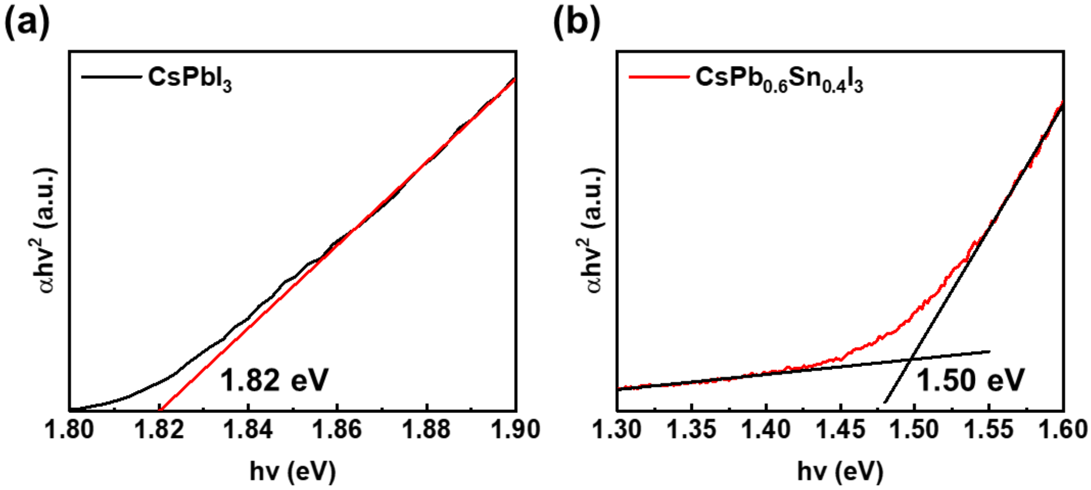

Figure S2. Tauc plot of (a) $CsPbI_3$ and (b) $CsPb_{0.6}Sn_{0.4}I_3$.

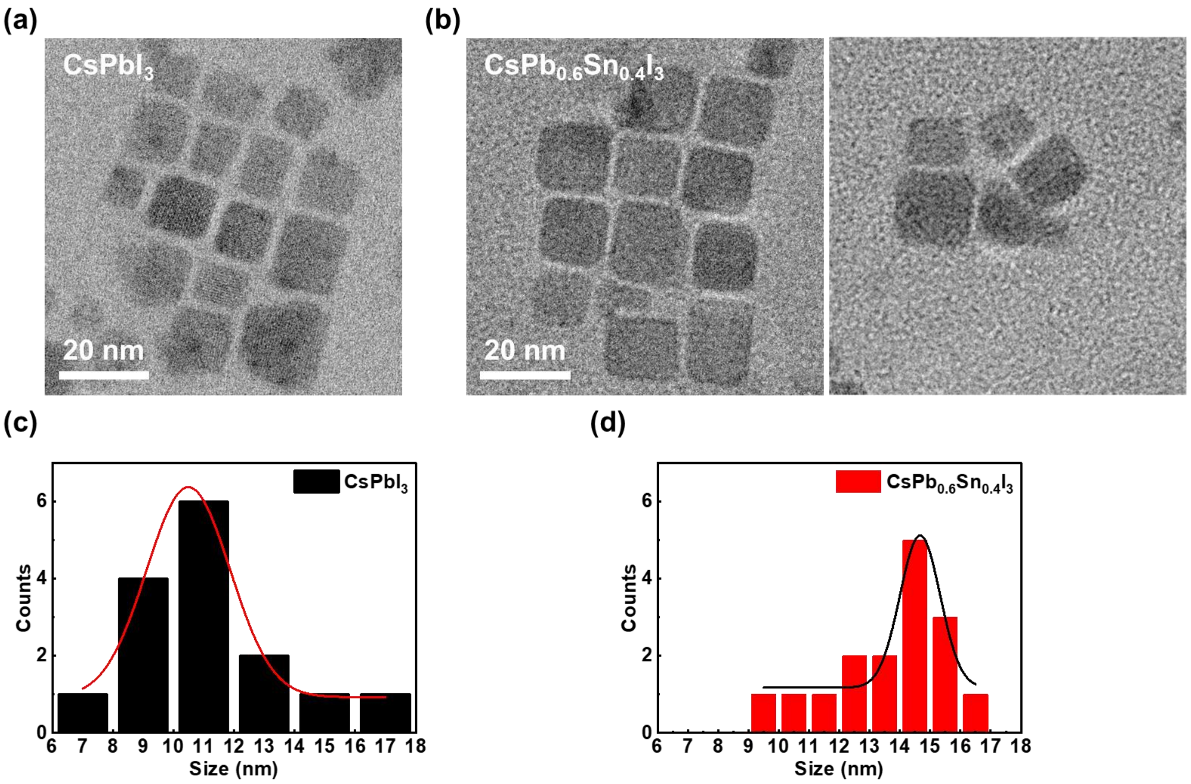

Figure S3. TEM images of (a) $CsPbI_3$ and (b) $CsPb_{0.6}Sn_{0.4}I_3$, and (c, d) size distribution, respectively.

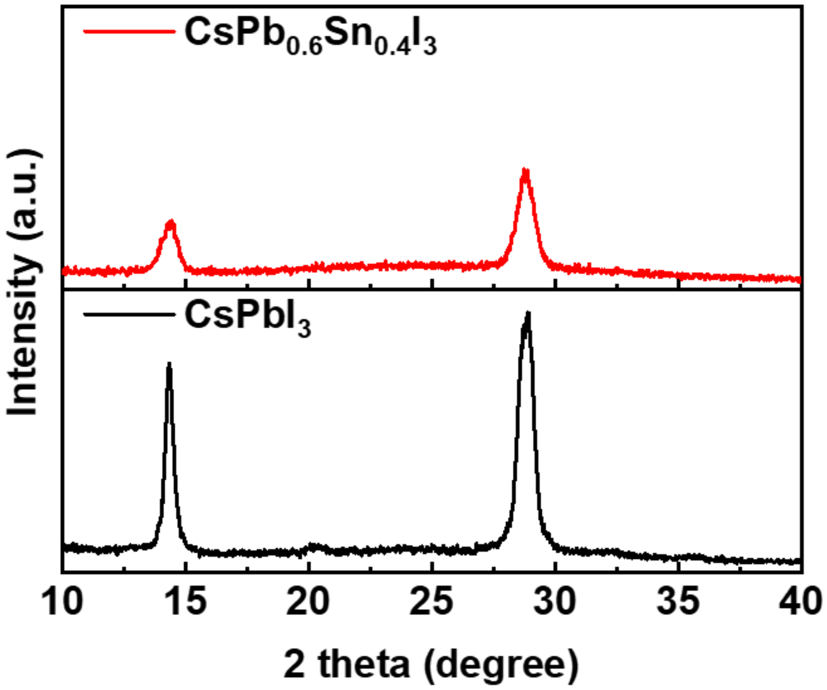

Figure S4. XRD of $CsPbI_3$ and $CsPb_{0.6}Sn_{0.4}I_3$.

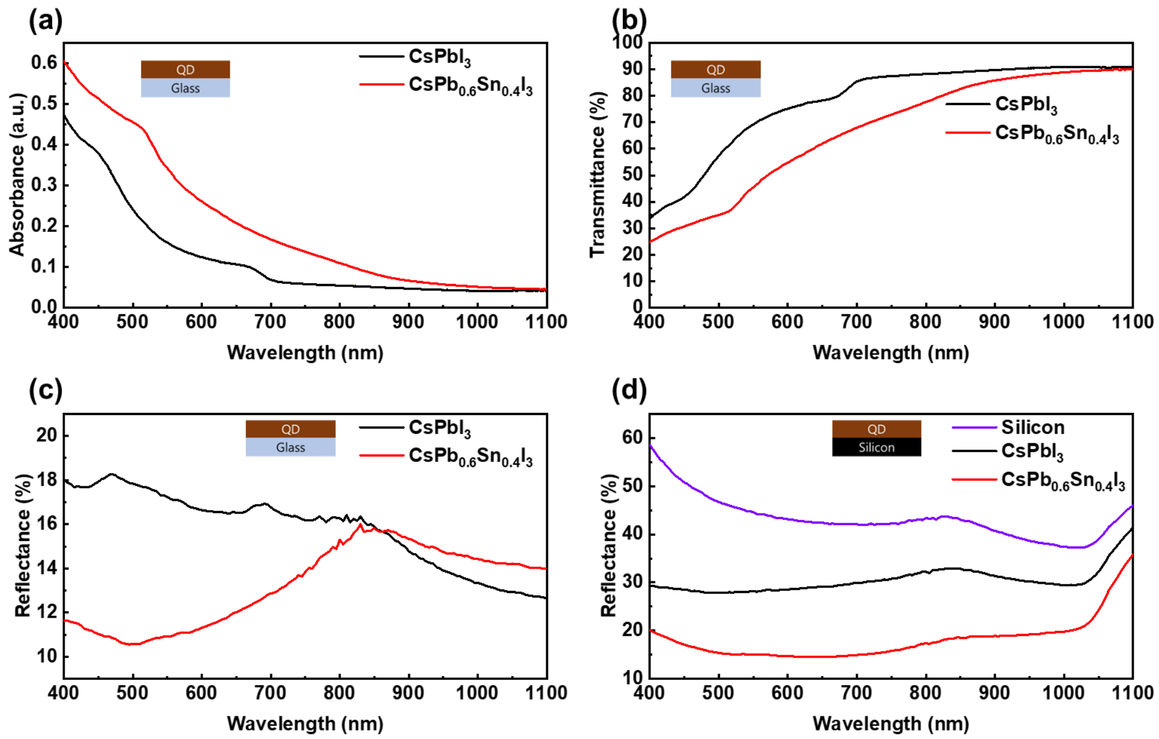

Figure S5. (a) Absorbance and (b) transmittance of the PQDs thin film on glass substrate. Reflectance of $CsPbI_3$ and $CsPb_{0.6}Sn_{0.4}I_3$ on (c) glass substrate and (d) silicon substrate.

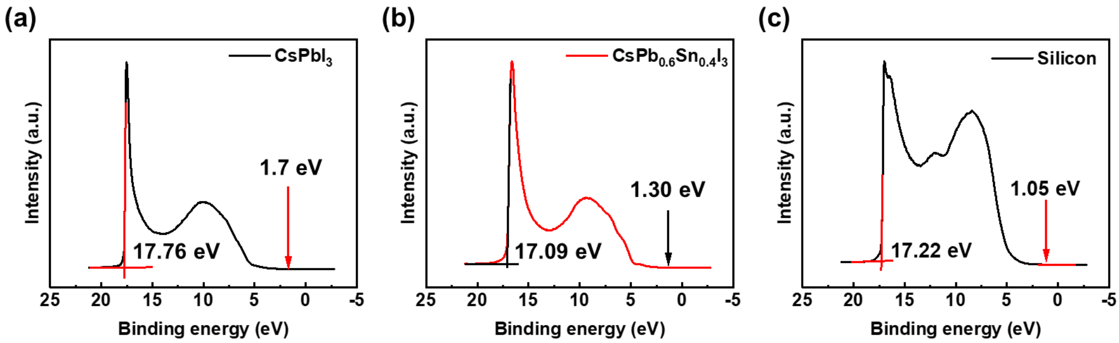

Figure S6. UPS of (a) $CsPbI_3$ and (b) $CsPb_{0.6}Sn_{0.4}I_{3,}$ and (c) n-type silicon.

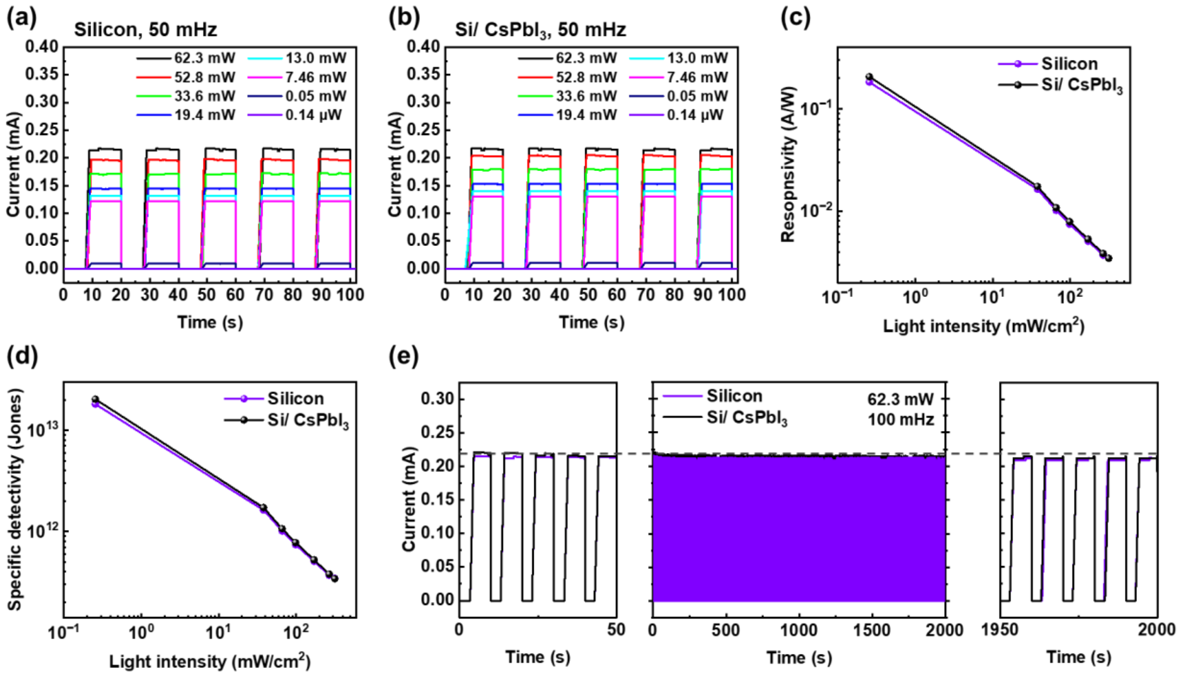

Figure S7. On/off current of (a) the silicon device and (b) Si/ $CsPbI_3$ under 1,064 nm light at 50 mHz. (c) Responsivity and (d) specific detectivity of each device. (e) 200-cycle on/off ratio of silicon device and Si/ $CsPbI_3$.

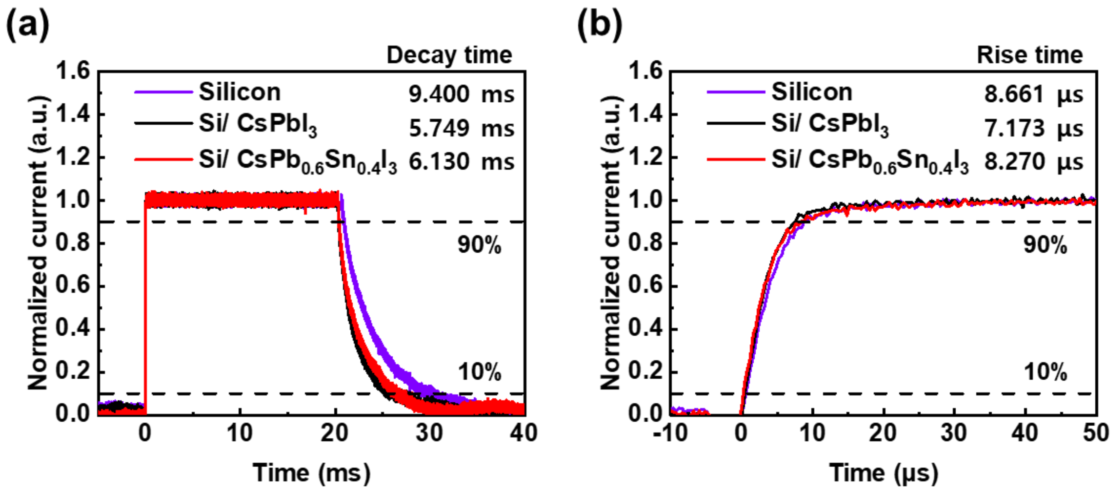

Figure S8. On/off ratio under 1,064 nm at 25 Hz. (a) Decay time and (b) rise time of each device.

Table S1. Atomic ratio of PQDs calculated by EDS.

No.	Element	Wt%	Wt% Sigma, σ	Atomic %	Ratio of Pb to Sn
1	Pb	6.42	0.31	1.16	0.65
	Sn	1.93	0.19	0.61	0.35
	Si	68.72	0.40	91.54	-
	Cs	5.31	0.28	1.50	-
	I	17.62	0.27	5.20	-
	Total:	100.00	-	100.00	-
2	Pb	9.32	0.34	1.98	0.63
	Sn	3.09	0.20	1.14	0.37
	Si	54.68	0.37	85.58	-
	Cs	6.79	0.32	2.24	-
	I	26.12	0.31	9.05	-
	Total:	100.00	-	100.00	-
3	Pb	17.12	0.80	11.26	0.58
	Sn	7.02	0.52	8.06	0.42
	Cs	16.37	0.80	16.78	-
	I	59.50	0.92	63.90	-
	Total:	100.00	-	100.00	-
4	Pb	17.40	0.73	11.45	0.57
	Sn	7.47	0.47	8.58	0.43
	Cs	16.11	0.75	16.53	-
	I	59.02	0.85	63.43	-
	Total:	100.00	-	100.00	-
5	Pb	17.62	0.74	11.61	0.58
	Sn	7.37	0.47	8.47	0.42
	Cs	15.56	0.76	15.98	-
	I	59.46	0.86	63.94	-
	Total:	100.00	-	100.00	-
